# Effects of Multi-Component Backgrounds of Volatile Plant Compounds on Moth Pheromone Perception

**DOI:** 10.3390/insects12050409

**Published:** 2021-05-01

**Authors:** Lucie Conchou, Philippe Lucas, Nina Deisig, Elodie Demondion, Michel Renou

**Affiliations:** Institute of Ecology and Environmental Sciences of Paris, iEES-Paris, INRAE, Sorbonne Université, CNRS, IRD, UPEC, Université Paris Diderot, 78026 Versailles, France; lucie@agriodor.com (L.C.); philippe.lucas@inrae.fr (P.L.); ndeisig@uni-koeln.de (N.D.); elodie.demondion@inrae.fr (E.D.)

**Keywords:** pheromone, plant volatile compounds, odor background, olfactory neuron, olfactory coding, odorscape, moth

## Abstract

**Simple Summary:**

It is well acknowledged that some of the volatile plant compounds (VPC) naturally present in insect natural habitats alter the perception of their own pheromone when presented individually as a background to pheromone. However, the effects of mixing VPCs as they appear to insects in natural olfactory landscapes are poorly understood. We measured the activity of brain neurons and neurons that detect a sex pheromone component in a moth antenna, while exposed to simple or composite backgrounds of VPCs representative of the odorant variety encountered by this moth. Maps of activities were built using calcium imaging to visualize which brain areas were most affected by VPCs. In the antenna, we observed differences in VPC capacity to elicit firing response that cannot be explained by differences in stimulus intensities because we adjusted concentrations according to volatility. The neuronal network, which reformats the input from antenna neurons in the brain, did not improve pheromone salience. We postulate that moth olfactory system evolved to increase sensitivity and encode fast changes of concentration at some cost for signal extraction. Comparing blends to single compounds indicated that a blend shows the activity of its most active component, VPC salience seems more important than background complexity.

**Abstract:**

The volatile plant compounds (VPC) alter pheromone perception by insects but mixture effects inside insect olfactory landscapes are poorly understood. We measured the activity of receptor neurons tuned to Z7-12Ac (Z7-ORN), a pheromone component, in the antenna and central neurons in male *Agrotis ipsilon* while exposed to simple or composite backgrounds of a panel of VPCs representative of the odorant variety encountered by a moth. Maps of activities were built using calcium imaging to visualize which areas in antennal lobes (AL) were affected by VPCs. We compared the VPC activity and their impact as backgrounds at antenna and AL levels, individually or in blends. At periphery, VPCs showed differences in their capacity to elicit Z7-ORN firing response that cannot be explained by differences in stimulus intensities because we adjusted concentrations according to vapor pressures. The AL neuronal network, which reformats the ORN input, did not improve pheromone salience. We postulate that the AL network evolved to increase sensitivity and to encode for fast changes of pheromone at some cost for signal extraction. Comparing blends to single compounds indicated that a blend shows the activity of its most active component. VPC salience seems to be more important than background complexity.

## 1. Introduction

Olfactory communication is essential to insects as it is involved in the identification and the location of vital resources such as a food source, a mate, or an oviposition site. Insects have developed an exquisite olfactory sense in terms of sensitivity, specificity, and temporal dynamics. Their olfactory system enables them to discriminate the pheromones they produce, as well as the odors involved in interspecific interactions, such as, for instance, the floral compounds emitted by plants to attract specialist pollinators. Herbivorous species, for instance, can discriminate potential host-plant species based on their volatile emissions [1]. Exchanges of chemical information are thus not only vital to insects, but also essential to the functioning of the species network composing a community [2]. Once released in the atmosphere, these ecologically relevant signals and cues are transported by airflows, diluted, and mixed to a background of other volatile organic compounds to form a complex and changing olfactory landscape [3]. Considering the hundreds of different volatile compounds released by plants (VPC) [4,5], the capacity of the insect olfactory system to extract the ecologically relevant information from that very complex chemical environment is remarkable [6,7].

Insect olfactory systems evolved to deal with such complex olfactory landscapes [8]. Male moths for instance are able to detect from hundreds of meters the odor plume generated by a female emitting its sex pheromone and to navigate upwind toward the calling female. Female moths release a few ng per hour of a specific pheromone blend, which represent only traces compared to the ppb of VPCs present in the atmosphere [9,10]. In the male antennae, narrowly tuned olfactory receptors (OR) expressed in olfactory receptor neurons (ORN) specifically bind the pheromone components [11,12] ensuring detection selectivity. The antennae house thousands of ORNs each of them expressing one functional type of OR specialized in the detection of one pheromone component (Ph-ORN). Beside quality, the firing activities of Ph-ORNs also encode the intensity of the stimulus. Ph-ORNs converge onto a comparatively small number of central neurons in a specialized area of the antennal lobes (AL), the macro-glomerular complex (MGC) [13]. Because of this convergence, the projection neurons (PN) in the MGC display a remarkably low response threshold [14,15,16]. Male moths not only discriminate the pheromone components, but also show ratio selectivity [17] which increases the specificity of pheromone communication. Blend ratio coding starts in the MGC, some MGC neurons responding more to blend of pheromone components than individual components [18,19]. Additionally, fast fluctuations of pheromone concentration are tracked by the ORN and MGC-neurons firing [20,21,22,23]. 

Odors within the habitat, and most specifically the volatile emissions from host-plants, affect moth behavioral responses to pheromone. Host plant volatiles, for instance, reduce the responses of *Spodoptera littoralis* males to deficient or heterospecific pheromone signals in a wind tunnel [24]. Plant and pheromone signals are processed by two anatomically distinct olfactory sub-systems, MGC and ordinary glomeruli (OG), but paired stimulations with a VPC and pheromone suppress responses in both MGC and OGs, indicating that both stimuli are not integrated independently [25]. Interactions can take place at the detection level and single VPCs modulate pheromone responses in male moths when presented together with pheromone. Effects on pheromone detection vary according to moth species and VPCs. Linalool and (Z)-3-hexenol synergize the responses of the Ph-ORNs of *Heliothis zea* to its pheromone [26]. Heptanal, a major component of the floral aroma of linden, activates Ph-ORNs of the noctuid moth *Agrotis ipsilon* [27]. However, other investigations report antagonistic interactions. Linalool decreases the responses of Ph-ORNs to pheromone compounds in *S. littoralis* [28]. A background of VPCs also modulates the firing of MGC neurons masking the response to pheromone [29]. Activity maps obtained by calcium imaging revealed intense MGC response to VPCs and various modes of interactions between pheromone and VPCs when they are presented together [30]. Interestingly, heptanal modified the multiphasic response-pattern of MGC-neurons to pheromone, decreased the response, but improved their capacity to encode pulsed stimuli [31,32]. The response to the pheromone component codlemone was suppressed in some AL neurons of male codling moths [33]. There is also evidence that some interactions take place at the OR level. Competitive fluorescence binding assays confirmed that plant odorants compete with the natural pheromone component, Z11 hexadecenal, for binding on HR13, a pheromone receptor of *Heliothis virescens* males [34]. A sex pheromone receptor of the codling moth, *Cydia pomonella*, also binds the plant volatile pear ester [35].

The ecological importance of VPC-pheromone interactions in natural conditions was recently questioned [36] because, although pheromone attraction of *H. virescens* males was significantly impaired in a concentration-dependent manner after adding single VPCs, their pheromone-guided flight behavior was not influenced by the natural emissions of host-plants in a wind tunnel. Badeke et al [36] concluded that the pheromone-VPC interactions only occur at supra-natural concentrations of VPCs. However, natural odor backgrounds are made of dozens of different compounds released by communities of host or non-host plants whose effects on pheromone communication could combine each to other. Furthermore, insect olfactory communication is now challenged by very fast and intense changes in VPC quality and concentrations notably due to the profound changes in land use and because plant metabolism is sensitive to global warming and increasing concentrations of CO_2_ and O_3_, which modifies the amounts of VPCs they release in the atmosphere [37,38]. Due to these changes in the olfactory landscapes in which insects live, it becomes crucial to better understand how multi-component olfactory backgrounds impact insect communication. Because of the preeminent role of sex pheromones in insect reproduction, pheromone-VPC interactions are a good model to address these questions. 

The present research aimed to investigate the effects of multi-component VPC backgrounds on pheromone perception in the moth *Agrotis ipsilon* (Hufnagel). The black cutworm, *A. ipsilon*, is a polyphagous and cosmopolitan moth belonging to the Noctuidae family that causes economic losses to many crops around the world. As in most moth species, pheromone communication is crucial for *A. ipsilon* mating, but males also rely on plant volatiles as food cues [39,40]. *A. ipsilon* has become a model species for studying the processing of pheromone and plant signals and their interaction at neuronal and behavioral levels [30,32,41]. Thus, this moth offers well-studied behavioral and physiological backgrounds to study the processing of plant-pheromone mixtures. The pheromone blend released by female *A. ipsilon* consists of three main components: cis-7-dodecenyl acetate (Z7-12:Ac), cis-9-tetradecenyl acetate (Z9-14:Ac), and cis-11-hexadecenyl acetate (Z11-16:Ac) [41,42,43]. Three functional types of pheromone sensitive ORNs (Ph-ORNs), each specifically tuned to either Z7-12:Ac, Z9-14:Ac, or Z11-16:Ac, have been identified in male antennae [44,45]. Previous investigations of their distribution along the antenna have shown that trichoid sensilla of the antennal branches house almost exclusively Ph-ORNs tuned to Z7-12:Ac (Z7-ORN). Z9-14:Ac-tuned ORNs (Z9-ORN) are less numerous and found only at branch tips [44]. Only two out of a sample of 100 neurons were found to respond to Z11-16:Ac [15]. A 3D glomerular atlas of the male antennal lobes has been established [46]. We used extracellular electrophysiology and calcium imaging to measure the responses of Z7- and MGC-neurons to the sex pheromone in simple or composite backgrounds of VPCs. Maps of activities were built using calcium imaging to visualize which VPCs activated areas in moth antennal lobes. To stimulate the moth antennae, we used a protocol that approximates the expected natural olfactory landscape in which a pheromone puff must be detected against a more diffuse odor background [27,29]. Accordingly, short pheromone puffs were delivered over long-lasting VPC backgrounds. We chose a panel of VPCs with different chemical structures and physicochemical properties representative of the odorant variety encountered by a moth searching for a mate. We first clarified the dose-response effects using single VPCs to create the background. A significant effort was paid to improve the control of stimulus intensity and to establish dose-response relationships. We evaluated the intrinsic activity for each VPC and determined their type of interaction with pheromone. We compared impact of VPC background at antennal and AL levels. Then, we prepared binary, ternary, and quaternary blends of VPCs to investigate interactions between VPCs and to determine whether properties of the blend could be deduced from the properties of single VPCs. Our data confirm that common VPCs interfere with the moth pheromone system in a dose-dependent manner. Activity varies among VPCs. The activity of a blend reproduces that of the most active component with only few interactions between components. We believe our data will contribute to better evaluate the vulnerability of insect olfaction to the ongoing changes in their olfactory landscapes. 

## 2. Materials and Methods

### 2.1. Insects

*A. ipsilon* adult males were obtained from a laboratory stock. Larvae were fed on an artificial diet [47]. Pupae were sexed and males were kept separately from females in an inversed light–dark cycle (16 h:8 h light:dark photoperiod) at 22 °C. This ensured that the males were virgin and had never been in contact with pheromone before experiments. Newly emerged males were collected every day and were given access to a 20% sucrose solution *ad libitum*. Day of emergence was considered as day-0. Males were aged of four days at experiment time. Experiments were performed during scotophase hours but under day light.

### 2.2. Chemicals

The pheromone component Z7-12Ac (CAS 14959-86-5), was purchased from Pherobank (purity > 99%). Based on the literature, we selected eight VPCs with different chemical structures and physicochemical properties to be representative of the variety of odorants that can be encountered by a male moth searching for a mate in an agricultural landscape in mainland France. The monoterpene linalool, the aromatic heterocyclic indole, and the bicyclic sesquiterpene β-caryophyllene have been identified in constitutive and herbivore-induced emissions of *Zea mais*, one of the host crop plants of *A. ipsilon* [48]. α-pinene is a common monoterpene, emitted by oak and other perennial species that grow on field edges [49]. The unsaturated hydrocarbon isoprene is one of the most abundant VPC in the atmosphere [50]; it is released among others by poplars planted to create windbreak hedges or for wood production [51]. (Z)-3-hexenyl acetate and (E)-2-hexenal are green leaf volatiles (GLV) produced by numerous plant species in response to biotic or abiotic stress [52]. Eucalyptol is a cyclic monoterpene released by flowering weeds, among which *Artemisia annua*, a common weed in maize fields [53]. VPCs were diluted in light mineral oil (CAS 8042-47-5). VPC synthetic standards (Table S1) and mineral oil were obtained from Sigma–Aldrich (Saint Louis, MO, USA).

### 2.3. Odor Stimulus Delivery

The stimulus delivering device consisted of an ensemble of electrovalves enabling to deliver VPCs from separate sources in the main air stream of a glass tube (length 200 mm, inner diameter 9 mm) whose distal end was positioned 20 mm from the insect antenna, while the pheromone stimuli were delivered through a lateral input (Figure S1). Air was charcoal-filtered and humidified.

Pheromone stimuli were delivered as air puff (167 mL/min) through a Pasteur pipette containing a piece of filter paper loaded with 10 ng (unless mentioned) of Z7-12:Ac diluted in 1 µL of hexane. Hexane was left to evaporate for 30 s before inserting the pheromone-loaded paper into the pipette. Air passage through the pipette was commanded by an electrovalve (LHDA1233215H, The Lee Company, Westbrook, ME, USA). The pipette tip was inserted into a hole on the side of the glass tube, 150 mm upstream of its distal end. 

VPC sources consisted of 4 mL glass vials containing 1 mL of a single VPC diluted in mineral oil, or mineral oil only (control). The air stream was divided into 8 parallel flows (200 mL/min each) with an airflow divider (LFMX0510528B, The Lee Company), each of which directed towards a 3-way electrovalve (LHDA1223111H, The Lee Company). Normally opened (NO, non-odorized) and normally closed (NC, odorized) exits of the eight valves were connected either to empty vials or to VPC source vials, respectively. All outlets of odorized and non-odorized vials were connected to the proximal end of the glass tube. Thus, valve opening did not modify the total airflow received by the antenna (1.6 L/min). All tubing downstream from the valves was made of Teflon (internal diameter 1.32 mm). Vials were connected to the tubing with stainless steel hypodermic needles inserted through a Teflon septum. For delivering single VPCs, the Teflon tubes at vial outlet were directly connected to the main glass tube (Figure S1A). VPC mixing was achieved by opening several valves simultaneously and mixing odorized airflows in a low dead-volume manifold (MPP-8, Warner Instruments, Figure S1B). For each of the 8 valves, the NO and the NC exits were connected together before entering one of the 8 manifold inlets. Temperature in the experimental rooms was regulated at 21–23 °C. The VPC and pheromone sources were allowed to equilibrate at the temperature of the rooms in which the experiments were carried out. 

### 2.4. Odor Stimuli

**Figure 1:**
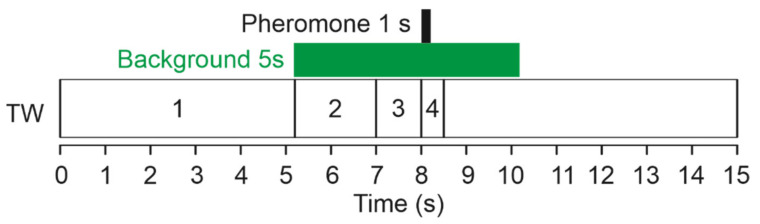
Stimulation protocol for electrophysiological experiments on Z7-ORNs and MGC-neurons. Black and green boxes indicate, respectively, the delivery of the background (VPC in mineral oil or pure mineral oil) and the pheromone compound Z7-12:Ac on the moth antenna. TW1 to TW4: limits of the time windows used to measure spontaneous activity (TW1), firing response to background (TW2), firing activity immediately before pheromone stimulus (TW3) and response to Z7-12:Ac (TW4).

Stimulation sequences consisted of a short pheromone puff delivered in the middle of a 5 s VPC background presentation (Figure 1). For electrophysiological recordings, the pheromone puff lasted 200 ms and started 2.8 s after background onset (Figure 1). For calcium imaging, the slow response dynamics of the fluorescence signal required to adapt stimulus duration. The pheromone puff lasted 1 s and started 2 s after background onset. Successive stimuli on the same preparation were separated by 30 s (antennal lobe recordings) or 60 s (single sensillum recordings and calcium imaging). Valve opening and closing sequences were computer-controlled with millisecond accuracy. The VPC were presented in random order, except for calcium imaging. In calcium imaging, stimuli with VPCs as a background to pheromone were alternated with stimuli with VPCs alone.

First, we evaluated the effects of single VPCs. Linalool, whose ability to activate the MGC-neurons of *A. ipsilon* had been already demonstrated [29], was used to establish the dose used for all VPCs of the panel. We first measured the activity of different dilutions of linalool on MGC-neurons, showing that a dilution of 1% triggered a clear firing activity. We adjusted dilutions of the other VPCs in function of their differences of volatility according to procedures proposed by Munch et al. [54] based on data from [55] (Table S1). The same dilutions were used for the sources of background in electrophysiological and Calcium imaging experiments.

Then, the effects of binary blends of VPCs and their components alone were tested at 4 concentrations each, while the pheromone dose was kept constant. In order to control precisely the ratios in the background, we measured their air–mineral oil partition coefficients (*K_hl_*, Table S1) and used them to calculate the mineral oil concentration (*C_l_*) necessary to obtain the desired concentration in the headspace (*C_h_*) of a closed, equilibrated source:

(1) $$C_{h}=K_{hl}*C_{l}\;\;\;$$

Partition coefficients *K_hl_* were measured by injecting the headspace of closed equilibrated sources containing known concentrations of VPCs into a calibrated GC-FID. When an airflow passes through such a source, the concentration in the headspace drops to a fraction of the initial concentration and then reaches a steady state until the source starts to exhaust. Therefore, odorant concentration delivered from the source is related to the initial concentration inside the closed equilibrated source by a dilution factor that does not depend on the odorant. Assuming that this proportionality remains true for the concentrations delivered on the antenna (i.e., no bias induced by differential adsorption on tubing walls), we expressed all delivered VPC concentrations relative to an arbitrary unit (AU) where one AU was defined as the molar concentration delivered on the antenna from a source containing 1% linalool in mineral oil. 

Binary blends were obtained by opening simultaneously the valves controlling the airflow through the sources containing the single VPCs to be blended while closing a compensation flow. Therefore, if the concentrations of blend components, when tested separately, are noted A and B, the blend was tested at the concentration A + B. The order of presentation of individual VPCs and of their combination was randomized. For each background type, 4 successive stimuli were presented at increasing concentration. A pheromone puff with no background was presented as a control at the beginning of the recording and after the highest concentration of each background type. We verified with a photoionization detector (PID; Aurora Scientific Inc, Aurora, Canada) that the concentration of VPC did not vary depending on whether it was delivered alone or in a multicomponent blend, as well as along successive stimuli separated by 5 or 10 min, with the exception of the very first stimulus (Figure S2). Experiments began by ejecting a VPC stimulus from all sources away from the recorded antenna. Then, the glass tube was focalized on the antenna and intervals between successive openings of the same VPC valve were always 5 or 10 min.

Finally, we tested blends of 3 or 4 VPCs as backgrounds to the pheromone stimulus. We alternated presentation of these complex backgrounds with backgrounds consisting in a single VPC or a binary blend of its components. Each of these 2 composite backgrounds and the corresponding simple backgrounds were presented twice to each neuron in random order. Control stimuli (pheromone without background) were presented at the beginning of each recording and after each pair of composite and simple background stimuli.

### 2.5. Electrophysiology 

For single sensillum recordings, male moths were briefly anesthetized with CO_2_ and restrained in a Styrofoam holder. One antenna was immobilized with adhesive tape. A tungsten electrode was inserted into the antenna to serve as a reference. We targeted the ORNs tuned to the pheromone constituent Z7-12:Ac (Z7-ORN) which are housed in the long trichoid sensilla located along antennal branches. The recording electrode was, therefore, inserted at the base of one of these sensilla. 

For extracellular recordings from MGC-neurons, male moths were restrained in a cut pipette tip, leaving the head exposed, and immobilized with dental wax. The head capsule was opened, and the brain exposed by removing all muscles and mouthparts above it. The neurolemma was carefully removed from the antennal lobe in order to allow electrode penetration. The recording electrode was made from a glass micropipette whose tip was manually broken to a diameter of 2 μm and filled with (in mM): NaCl 150, KCl 4, CaCl_2_ 6, MgCl_2_ 2, Hepes 10, Glucose 5 (pH 7.2, osmotic pressure 360 mOsm/L adjusted with mannitol). The preparation was constantly perfused with this solution once the brain capsule was opened. The reference electrode was a silver wire inserted at the rear of the head capsule in contact with brain tissues. The recording electrode was slowly inserted inside the MGC until the appearance of a clear single-unit firing activity. Extracellular recordings from *A*. *ipsilon* AL sample only neurons with a large neurite [16] so we expected to record mainly projection neurons (PN) rather that local interneurons (LN). Recordings were done using a CyberAmp 320 controlled by pCLAMP10 (Molecular Devices, San Jose, CA, USA). The biological signal was amplified (×2000), band-pass filtered (1–3000 Hz) and sampled at 10 kHz with a Digidata 1440A acquisition board (Molecular Devices). Spikes were sorted using Spike 2 software (CED, Oxford, UK).

### 2.6. Calcium Imaging in Antennal Lobes

Male moths were restrained in a Plexiglas chamber and the head was fixed. The head was opened and muscles and mouthparts removed to gain access to the brain. Then, 20 µL of a dye solution (50 µg Calcium Green 2-AM dissolved with 50 mL Pluronic F-127, 20% in dimethylsulfoxide, Molecular Probes, Eugene, OR, USA) was bath-applied for at least 1 h, before being washed with Ringer. For recordings, a T.I.L.L. Photonics imaging system (Martinsried, Germany) was coupled to an epifluorescent microscope (BX-51WI, Olympus, Hamburg, Germany) equipped with a 10× (NA 0.3) water immersion objective. Signals were recorded using a 640 × 480 pixel 12-bit monochrome CCD camera (T.I.L.L. Imago, cooled to −12 °C). The acquisition rate was set at 5 frames/s with an acquisition time of 15 ms. Identification of individual glomeruli was done by superposing activity maps using Adobe Photoshop (Version CS2). We chose not to include isoprene in calcium imaging experiments due to the limited number of channels available in the odor stimulation device.

Raw data analysis was done using custom–made software written in IDL (Research Systems Inc., Boulder, CO, USA) and Visual Basic (Microsoft Excel). After noise filtering and bleaching correction, relative fluorescence changes (δF/F) were calculated as (F − F_0_)/F_0_ (where F_0_ = reference background). For each glomerulus, the time course of δF/F was calculated by averaging 25 pixels (5 × 5) at the center of each glomerulus.

### 2.7. Analysis of Electrophysiological Data 

Analysis of firing activity was performed using custom programs developed under R [56]. An instantaneous firing rate metric [57] was used to draw peri-stimulus firing curves. For each individual recording, a firing rate was calculated for every spike using the two preceding and two following spikes. Then, we calculated the average firing rates over successive 75 ms-long time bin in each recording. Individual neuron firing rates/bin were finally averaged over all sampled neurons to draw peri-stimulus curves.

We calculated the maximum firing frequency within four time-windows (TW) covering the successive phases of the two stimuli. The limits of each TW are the valve opening times shifted to consider the travel time of the odorized airflows from the valves to the antenna (Figure 1). TW1 (from 0 to 5.2 s) covered the period before background application and was used to measure the spontaneous activity. The phase corresponding to VPC background onset until pheromone puff was split into two TWs. When a response to background was visible, TW2 (5.2–7.0 s) covered the rise in the neuron firing that followed the background onset; TW3 (7–8 s) covered the period during which the firing decreased compared to the peak, but stayed above the spontaneous activity; TW4 (8–8.5) covered the response to pheromone. To correct for differences in spontaneous activity between neurons, we calculated the **response to background** by subtracting the mean firing frequencies in TW1 (mean_TW1_) from the maximum firing frequency reached in TW2. Similarly, the **pheromone response** was calculated by subtracting mean_TW1_ from the maximum firing frequency reached within TW4 (max_TW4_). We estimated the capacity of neurons to extract the pheromone signal from the background, the **pheromone salience,** by subtracting pre-pheromone activity level in TW3 from max_TW4_. 

For experiments evaluating the effects of single VPCs we used pairwise paired t tests on all possible pairs of background types to compare values of response to background, response to pheromone and pheromone salience in the different backgrounds. False discovery rate was controlled using the Benjamin-Hochsberg’s procedure (FDR < 0.05). 

For experiments comparing blends to their components we used a permutational MANOVA (PERMANOVA, function Adonis()) under R package vegan [58,59] to evaluate how background composition and dose affected neuronal responses, the three measured variables taken together. The PERMANOVA used a Euclidian distance matrix calculated from response to background, response to pheromone, and pheromone salience. For significance testing, permutations were restricted within individual recordings (parameter “strata”) to account for the non-independence of observations made on the same neuron. Whenever significant differences were found, we further tested differences between the blend and each component using pairwise PERMANOVAs between all three pairs of background types. False discovery rate was controlled using the Benjamin-Hochsberg’s procedure (FDR < 0.05). Note that under this analysis, dose was considered a categorial variable, which may not be sufficient to accurately describe the way neurons respond to a blend of two agonistic components. Therefore, for the blend (Z)-3-hexenyl acetate/linalool we further refined the analysis by taking the actual concentrations into account through modeling the dose-response curves. Dose-responses to odorants are usually modeled using Hill’s equation [60]: the response is described as a function of ligand concentration (*C*), and depends on a maximal response intensity (*R_max_*), a concentration at half maximum (*EC*_50_), and Hill’s coefficient (*n*):

(2) $$Response=\frac{C^{n}*R_{max}}{C^{n}+EC_{50}^{n}}$$

Our response to background data were not appropriate for a fit of Hill’s equation because the saturation was never reached making it impossible to estimate *R_max_*. However, in the case of pheromone response and of pheromone salience, the aim was to model the decrease in response intensity observed in the presence of an agonist background. For that purpose, response intensity or salience in the absence of background could be considered as *R_max_*. Since individual neurons differed in their responsiveness to pheromone, we normalized all observed pheromone response and salience values to the considered neuron’s corresponding *R_max_*:

(3) $$R_{norm}=\frac{TW4-TW3}{R_{max}}\;\;$$

and

(4) $$S_{norm}=\frac{TW4-TW1}{S_{max}}$$

This also allowed to simplify Hill’s equation by setting *R_max_* = 1. Subtracting this simplified Hill’s equation from 1 produces a curve that decreases with increasing *C*, which is empirically appropriate for fitting on the observed decreasing response to pheromone or pheromone salience as function of the background dose:

(5) $$R_{norm}\;or\;S_{norm}=1-\frac{C^{n}}{C^{n}+EC_{50}^{n}}=\frac{EC_{50}^{n}}{C^{n}+EC_{50}^{n}}$$

We fitted Equation (5) using the non-linear regression function nls2() (R package nls2, G. Grothendieck, 2013, CRAN deposit) to estimate EC_50_ and *n* for the Z7-ORN recordings pooled together (*n* = 16), and for each background type separately, using total background concentration in arbitrary units as C. We compared the dose-responses observed under blended background and under single compound’s background by checking whether the confidence intervals for the fitted parameters did or did not overlap.

## 3. Results

### 3.1. Calcium Responses to Single VPCs in the MGC

**Figure 2:**
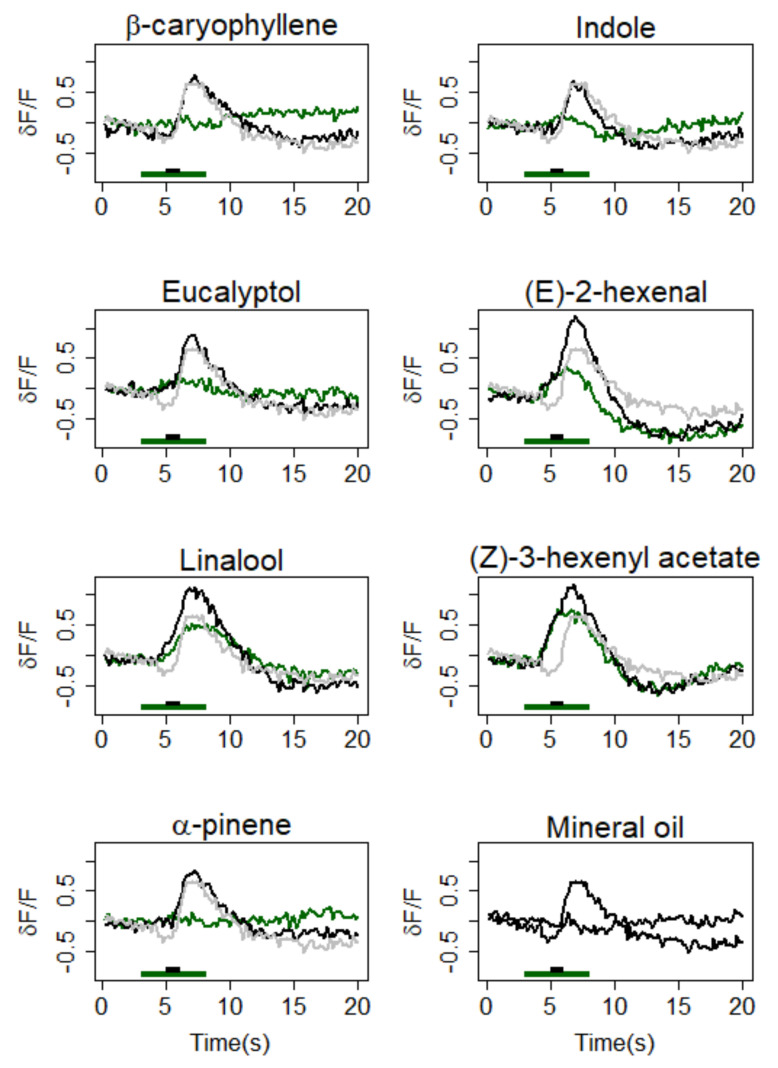
Effects of a VPC background on the calcium-response to pheromone in the MGC area of the antennal lobe. Average time course (*n* = 15) of δF/F in response to VPCs (green curves) and to the pheromone in a background of the same VPCs (black curves) and to pheromone in control background (grey curves). The last panel presents the responses to the pheromone in the control background (mineral oil only) and to the control background. Green and black bars at the bottom of each graph mark the background and pheromone stimuli, respectively.

Z7-12:Ac activated a large area in the AL that we identified as the MGC according to its position, similar to previous observations in the same insect species [30,61] (Figure S3). All tested VPCs induced a calcium response (Ca-response) in some areas of the antennal lobes corresponding to different ordinary glomeruli (Figure S3). Activity patterns differed according to VPCs, with (E)-2-hexenal, (Z)-3-hexenyl acetate, and linalool activating repeatedly large areas of the ALs, while β-caryophyllene, eucalyptol, and indole showed more limited activity maps (Figure S3). α-pinene elicited a very localized increase in fluorescence in only two preparations (Figure S3). (Z)-3-hexenyl acetate, linalool, and (E)-2-hexenal triggered a strong Ca-response within the MGC (Figure 2). The Ca-responses to Z7-12:Ac and VPCs were tonic, lasting the time of the odorant presentation (Figure 2). Eucalyptol, indole, caryophyllene, and α-pinene did not evoke a significant Ca-response in the MGC.

When pheromone was delivered during the VPC-background, Ca-responses to VPC and to Z7-12:Ac merged to form a single fluorescence-peak (Figure 2). Thus, it was not possible to quantify separately the contribution of the VPC and that of pheromone to the Ca-response. However, in the MGC, the Ca-response to VPC plus Z7-12:Ac was always significantly larger than that of VPC alone, independently of the VPC (Table 1, test background vs. background + Phe). It was significantly larger compared to pheromone in mineral oil for 2-hexenal, linalool, and Z3-hexenyl acetate, the three VPCs which triggered a Ca-response within the MGC (Table 1). For the other four VPCs of the panel, the Ca-response to Z7-12:Ac in a VPC background was not different from that to Z7-12:Ac in the control background. The response to Z7-12:Ac alone was always above that to the VPC alone, the difference was significant for α-pinene, Eucalyptol, Indole, β-caryophyllene (Table 1).

### 3.2. Some Single VPCs Activate MGC Neurons and Affect Their Responses to Z7-12:Ac

As the Ca-signal measured with bath applied calcium dye is generally considered as mainly reflecting the sensory input from ORNs, we undertook to record the firing activity of the MGC neurons. To specify at which VPC dose the MGC neurons are expected to respond, we first tested linalool, a compound previously shown to stimulate MGC neurons activity [29]. Overall, seven doses of linalool ranging from 0.00001 to 10% in mineral oil were tested for their effects on the firing activity and responses to pheromone in 5 MGC-neurons. It confirmed that linalool activates the firing of the MGC-neurons in a dose-dependent manner (ANOVA, *p* < 0.0001), partially masking the response to Z7-12:Ac. The lowest active dose was 1% (posthoc paired t-tests, compared to control: response to background *p* = 0.0631; response to pheromone, *p* = 0.0019; pheromone salience, *p* < 0.0001). Recordings showed a fast rise of the firing at the background onset, followed by a plateau lasting the time of the linalool presentation. Linalool 1% was chosen as a reference stimulus for further experiments.

**Figure 3:**
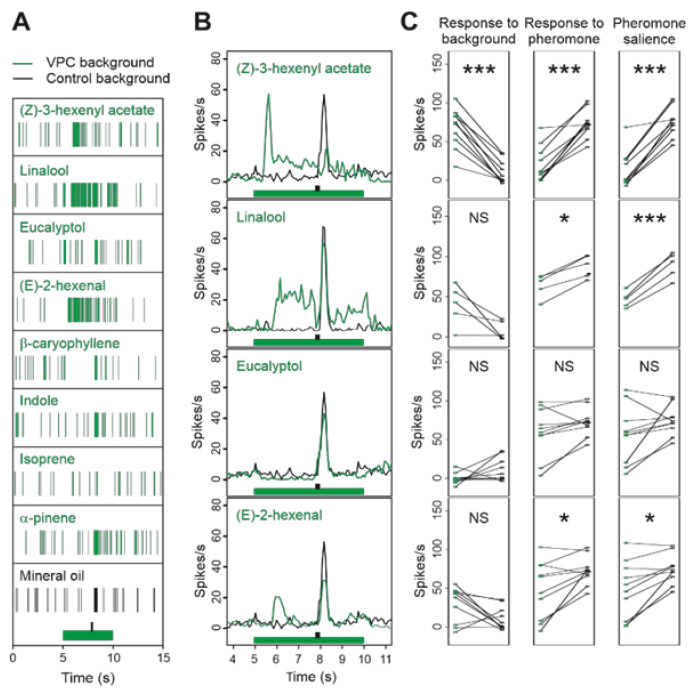
Effects of a VPC background on the firing activity of MGC-neurons and their responses to the pheromone. (**A**): raster plots of typical individual extracellular recordings. (**B**): Mean frequency plots showing the fast firing peak at background onset, followed by a sustained firing activity, and their effects on the amplitude of the firing peak at pheromone presentation. In (**A**,**B**), green and black bars indicate background and pheromone stimuli, respectively. (**C**): Strip charts comparing individual firing activities in each VPC background (green dots) with the control background (black dots). Firing frequency was measured on appropriate time windows to evaluate: response to background (left column), response to pheromone (middle column), and pheromone salience (right column). * and *** indicate *p*-values of the paired t test below FDR threshold; NS = *p*-value above FDR threshold. *n* = 5 for linalool and 10 for other compounds.

Then, we stimulated moth antennae with the other VPCs of the panel, adjusting their concentrations in the source vial according to their volatilities (Table S1). Responses of MGC neurons (*n* = 10) varied according to the VPC (ANOVA, *p* > 0.00001). (Z)-3-hexenyl acetate strongly stimulated the MGC-neurons (Figure 3A; posthoc t-test, response to background: *p* < 0.0001). Compared to linalool, responses to (Z)-3-hexenyl acetate were more dynamic, showing a fast initial peak with a short latency (Figure 3B), followed by a decrease in firing and a sustained plateau until background offset. Increases in firing were also observed in some recordings in response to (E)-2-hexenal (Figure 3A–C), but the difference with the control background is not significant (response to background: *p* = 0.0579) due to the variability of responsiveness of MGC-neurons to this VPC (Figure 3C). Several MGC-neurons showed a decrease in their firing activity at eucalyptol presentation (Figure 3C) suggesting an inhibitory activity but the difference with the control is not globally significant (response to background: *p* = 0.0928). 

Contrary to the Ca-imaging experiments, it was possible to isolate the increase in firing activity in response to the pheromone puff and to measure the pheromone salience. Response to pheromone was dramatically reduced in a (Z)-3-hexenyl acetate background (*p* < 0.0001), indicating the occurrence of mixture suppression and resulting in an almost complete masking of pheromone (Figure 3C). Reduction in the response to pheromone was also observed in the presence of (E)-2-hexenal background (*p* = 0.0169). The combination of response to VPC background and reduction in response to pheromone resulted in a significant reduction in pheromone salience in the presence of (E)-2-hexenal (*p* = 0.0061), linalool (*p* = 0.0003), and (Z)-3-hexenyl acetate (*p* < 0.0001) backgrounds. 

Although the firing activity seemed lower in the presence of eucalyptol in some MGC-neurons (Figure 3C), this decrease was not significant (*p* = 0.0579) when pooling neurons and response to pheromone and pheromone salience were not statically different between eucalyptol and control background. β-caryophyllene, indole, isoprene, and α-pinene neither elicited significant responses nor significantly altered the response to pheromone (Figure S4).

### 3.3. Single VPCs Modulate the Z7-ORN Spontaneous Firing and Affect Their Responses to Pheromone

**Figure 4:**
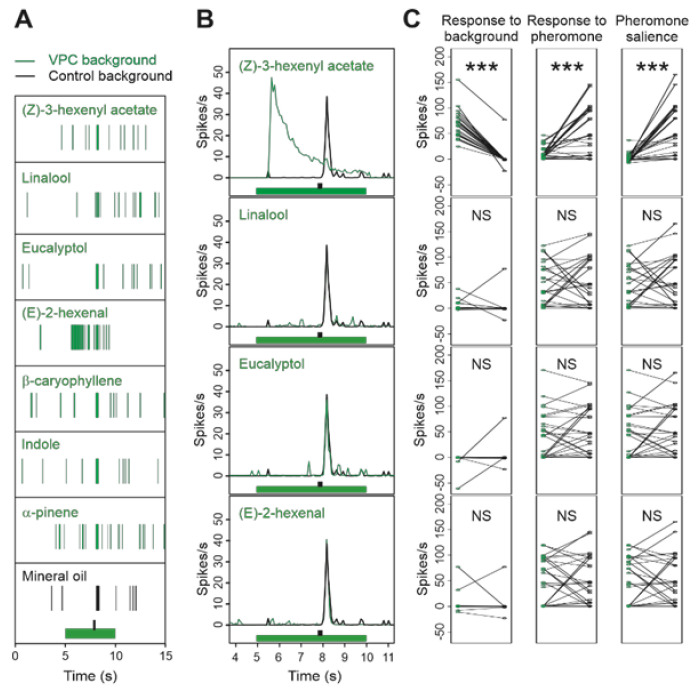
A background of (Z)-3-hexenyl acetate, linalool, eucalyptol or (E)-2-hexenal modifies the firing activity of Z7-ORNs and their responses to pheromone. (**A**): raster plots of samples of single sensillum recordings. (**B**): Mean frequency plots showing the time-course of the firing in response to background presentation and pheromone pulse. In (**A**,**B**), green and black bars indicate background and pheromone stimuli, respectively. (**C**): Strip charts comparing individual neuron firing activities in each VPC background (green dots) with the control background (black dots). Firing frequency was measured on appropriate time-windows to evaluate the response to background (left column), response to pheromone (middle column), and pheromone salience (right column). *n* = 26. Stars indicate *p*-values of the paired t test below FDR threshold; NS = *p*-value above FDR threshold. *n* = 5 for linalool and 10 for other compounds.

We then recorded the responses of Z7-ORNs to pheromone in the presence of the same VPCs and at the same concentrations as for MGC-neurons and compared the effects of VPC backgrounds on Z7-ORNs and MGC-neurons to clarify whether interactions took place at peripheral or AL levels. The impact of (Z)-3-hexenyl acetate background was similar on ORNs as it was on MGC-neurons (Figure 4). Z7-ORNs responded to the background with a significant increase in firing (*p* < 0.0001). However, compared to MGC-neurons, their firing activity decreased more slowly with a longer tail (Figure 4B). The response to pheromone was significantly reduced (*p* = 0.0004). The firing peak in response to pheromone was hardly visible (Figure 4B) and accordingly pheromone salience was strongly decreased (*p* < 0.0001) (Figure 4C).

Z7-ORNs did not respond to the linalool background (Figure 4B; *p* = 0.6724), unlike MGC neurons, nor to a (E)-2-hexenal background (*p* = 0.6709). Response to pheromone and pheromone salience were not altered by linalool (*p* = 0.5710 and 0.4598, respectively) or (E)-2-hexenal (*p* = 0.9933 and 0.9883, respectively). In agreement with its lack of activity in MGC-neurons, no significant effect of α-pinene was observed in Z7-ORNs, either for response to background (Figure S5; *p* = 0.5447), response to pheromone (*p* = 0.6244), or pheromone salience (*p* = 0.8908). The indole background did not activate Z7-ORNs (Figure 4 and Figure S5; response to background *p* = 0.73542), and did not modify response to pheromone (*p* = 0.8275) and pheromone salience (*p* = 0.1126).

To highlight putative inhibitions, we applied backgrounds as short pulses over a sustained pheromone stimulation (Figure S6). Compared to control, Z7-ORNs stimulated by pheromone had a lower activity during presentation of eucalyptol than before (Figure S6; *p* = 0.023 for the comparison of the difference in number of spikes in TW4 and TW3). Such inhibitory responses were not observed for (E)-2-hexenal (*p* = 0.0621), α-pinene (*p* = 0.254), indole (*p* = 0.9611), β-caryophyllene (*p* = 0.1920), and linalool (*p* = 0.8499). We also noted a significant increase for (Z)-3-hexenyl acetate (*p* = 0.0068), due to the response to the background.

Altogether, Ca-imaging and electrophysiology allowed to identify (Z)-3-hexenyl acetate, linalool, and to a lesser extent (E)-2-hexenal, as Z7-12:Ac agonists with masking activity of the pheromone responses at the peripheral or AL levels. Eucalyptol was postulated to be a weak inhibitor. The other VPCs, indole, α-pinene, β-caryophyllene and isoprene were postulated to be neutral at the tested concentration. In the next step we combined these VPCs in 2 to 4 component blends to determine whether they interacted at the level of Z7-ORNs.

### 3.4. Binary Blends Produce the Effects of Their Most Active Component

We prepared binary blends by combining VPCs having demonstrated contrasted impacts on the pheromone perception and compared their effects on Z7-ORNs to those of their individual components at different doses. To facilitate comparisons between single components and their blends the concentrations delivered to the antenna were expressed in arbitrary units (AU) as defined in the material and methods.

**Figure 5:**
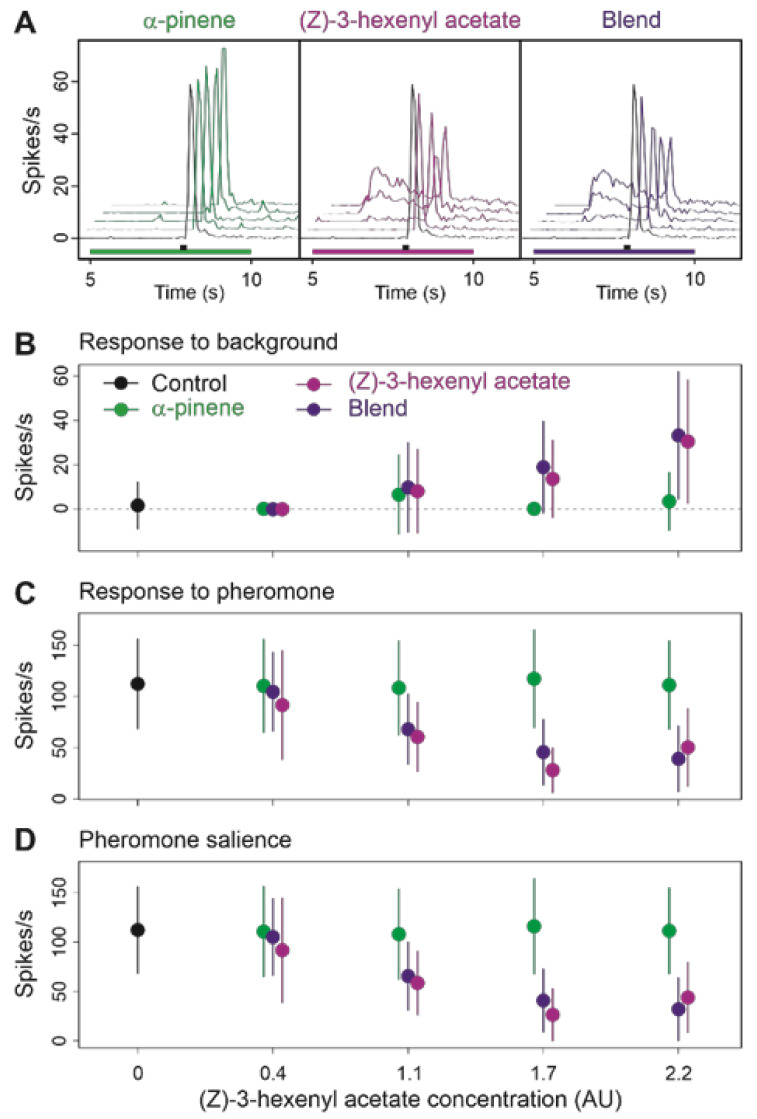
Effects on Ph-ORNs of a blend of α-pinene and (Z)-3-hexenyl acetate at 2:1 ratio as background to pheromone. (**A**): Mean frequency plots (*n* = 15) showing the time-course of the firing during background presentation and after pheromone pulse at increasing concentrations of background; below the plots green (α-pinene), light violet ((Z)-3-hexenyl acetate) or dark violet (blend) bars indicate background stimulus and black bars indicate pheromone stimulus. (**B**–**D**): Effects of background dose and composition on response to background (**B**), response to pheromone (**C**) and pheromone salience (**D**). Means and standard deviations, *n* = 15.

We first mixed the agonist (Z)-3-hexenyl acetate with α-pinene, which neither stimulated MGC-neurons and Z7-ORNs nor modified their response to pheromone, to determine whether the effect on pheromone response of a VPCs could be antagonized by another VPC. To increase the probability of evidencing an interaction between these two VPCs when blended we doubled the proportion of α-pinene relatively to that of (Z)-3-hexenyl acetate (ratio 2:1). The effects of (Z)-3-hexenyl acetate on Z7-ORNs were clearly dose-dependent (Figure 5; global PERMANOVA, dose effect *p* = 0.001) and responses to the background increased with the concentration of (Z)-3-hexenyl acetate (Figure 5A,B). The responses to pheromone were more strongly attenuated at higher concentrations (Figure 5C). Consequently, the pheromone salience decreased when increasing the background concentration (Figure 5D). The firing of Z7-ORNs was the same in a background of α-pinene as in the control background, whatever the α-pinene concentration (Figure 5A,B; pairwise PERMANOVA, α-pinene versus control backgrounds, *p* = 0.939). When the binary blend was presented as a background, neuron activities were not different from those observed under a background of (Z)-3-hexenyl acetate alone (Figure 5; pairwise PERMANOVA on (Z)-3-hexenyl acetate versus blend backgrounds, background type effect *p* = 0.262).

**Figure 6:**
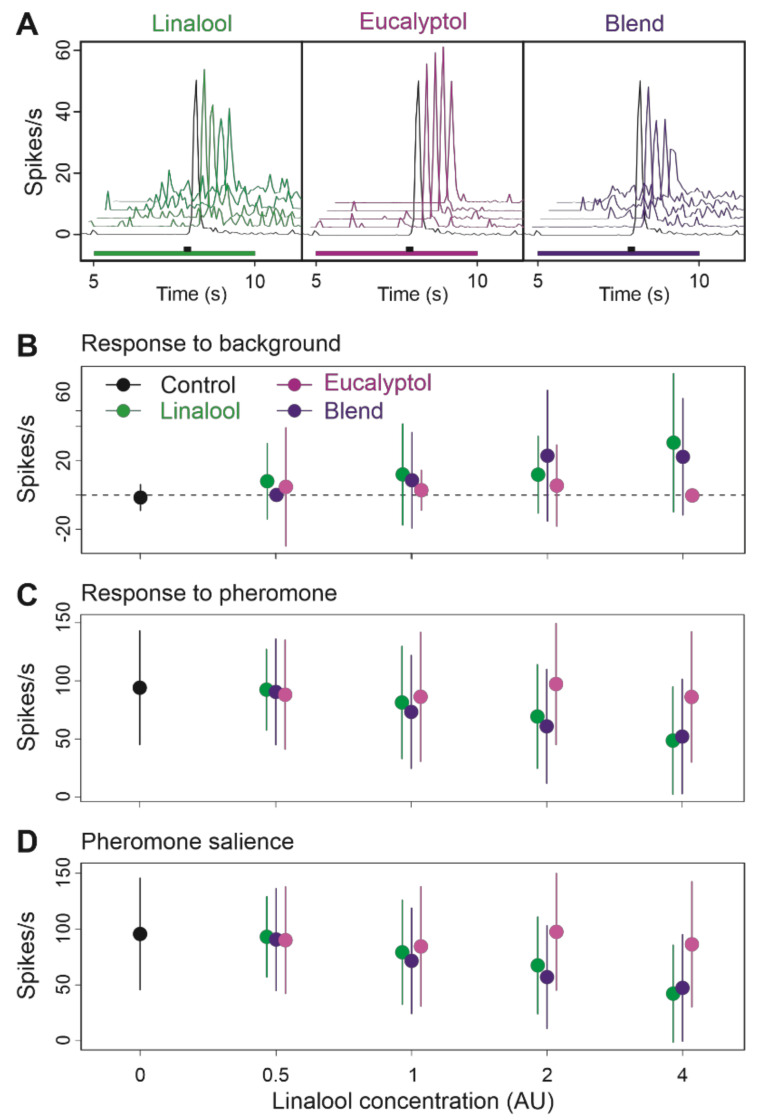
Effects on Z7-ORNs of blending linalool with eucalyptol at a 1:2 ratio as background to a pheromone stimulus. (**A**): Mean frequency plots (*n* = 17) showing the time-course of the firing during background presentation and after pheromone pulse; below each plot a green (linalool), light violet (eucalyptol) or dark violet (blend) rectangle indicates background presentation and a black rectangle the pheromone stimulus. (**B**–**D**): Effects of background dose and composition on response to background (**B**), response to pheromone (**C**), and pheromone salience (**D**). Means and standard deviations, *n* = 17.

Next, we tested a blend of linalool, which showed agonist activity at AL level and has been formerly reported to stimulate Z7-ORNs [27], with eucalyptol, a VPC that inhibited some MGC neurons and Z7-ORNs when presented over a sustained pheromone stimulation, at a ratio of 1:2 (Figure 6). At the tested concentrations, eucalyptol alone did not have any noticeable impact on the neuronal activity (Figure 6B; pairwise PERMANOVA eucalyptol vs. control backgrounds, background type effect *p* = 0.212). At high concentrations, linalool activated Z7-ORN, confirming a dose-dependent agonist activity at ORN level (global PERMANOVA, background type effect *p* = 0.001, dose effect *p* = 0.01). Response to the linalool background was apparent only at 4AU (Figure 6B) while the reduction in pheromone response and salience appeared from 2AU (Figure 6C,D). The blended background did not modify neuronal activity any differently from linalool alone (pairwise PERMANOVA linalool versus blended background, background type effect *p* = 0.570, Figure 6B–D) suggesting eucalyptol did not interact with linalool.

**Figure 7:**
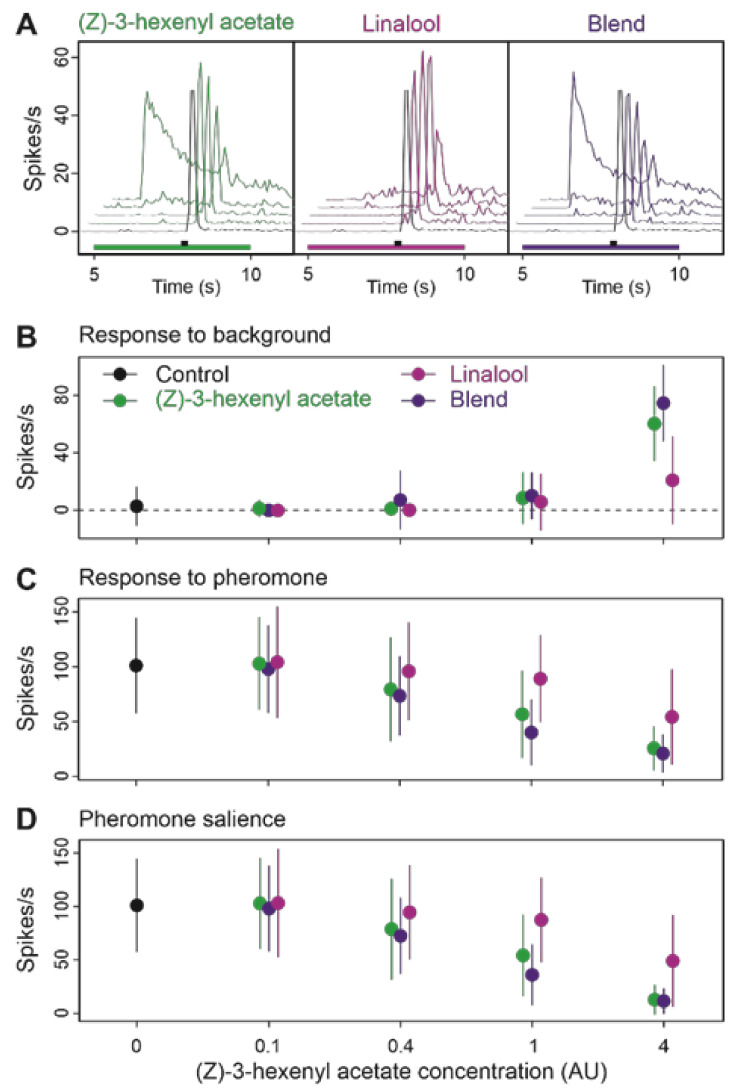
Effects on Z7-ORNs of blending two agonists VPCs, (Z)-3-hexenyl acetate and linalool, at a 1:1 ratio as background. (**A**): Mean frequency plots showing the time-course of firing during background presentation and after pheromone pulse. Below the plots, green ((Z)-3-hexenyl acetate), light violet (linalool) or dark violet (blend) rectangles indicate background presentation; a black rectangle indicates the pheromone stimulus. (**B**–**D**): Effects of background dose and composition on response to background (**B**), response to pheromone (**C**), and pheromone salience (**D**). Means and standard deviations, *n* = 16.

We then mixed the two agonists (Z)-3-hexenyl acetate and linalool at a 1:1 ratio and compared the blend effects to that of its individual components. Comparison of the activity of Z7-ORNs in response to increasing doses confirmed that linalool was a weaker agonist than (Z)-3-hexenyl acetate (pairwise PERMANOVA (Z)-3-hexenyl acetate versus linalool, background type effect, *p* = 0.0010; Figure 7A,B). Adding the two VPCs to each other resulted in a significantly stronger activity of the blend compared to linalool alone (pairwise PERMANOVA linalool versus blend, background type effect, *p* = 0.001). The blend was also more active than (Z)-3-hexenyl acetate alone at the two highest concentrations (pairwise PERMANOVA, (Z)-3-hexenyl acetate versus blend, background type effect *p* = 0.001). 

**Figure 8:**
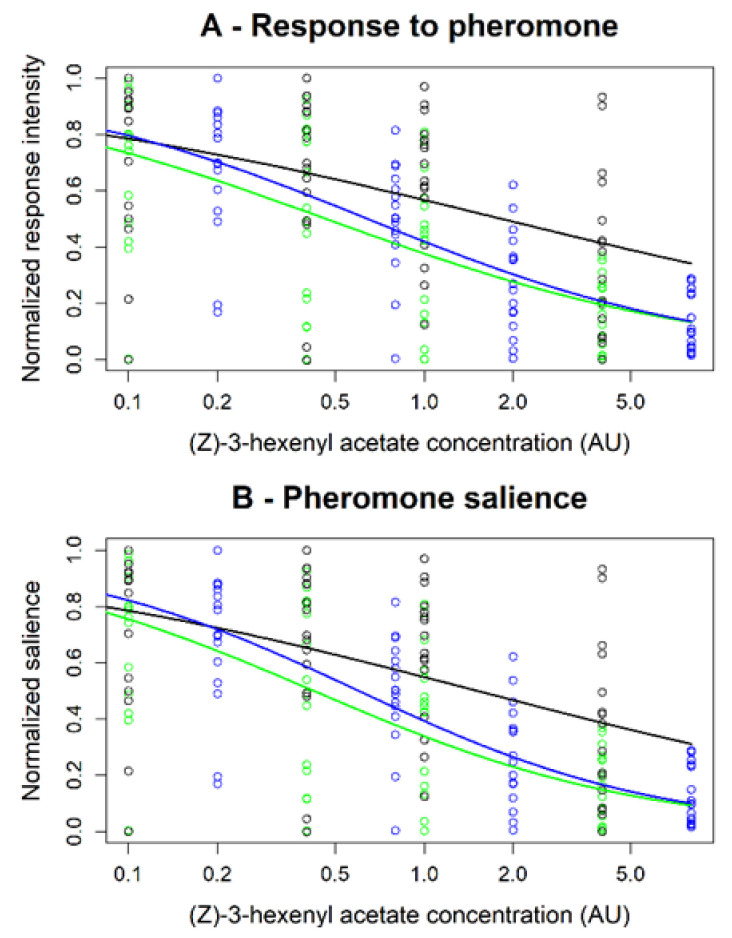
Modeling the dose dependence of the effects of blend (Z)-3-hexenyl acetate and linalool on (**A**) response to pheromone and (**B**) pheromone salience. Circles = experimental values after normalization in linalool (black dots), (Z)-3 hexenyl acetate (green dots), and the 1:1 blend (blue dots). Lines = predicted values obtained from fits of the modified Hill’s equation $R_{norm}\;or\;S_{norm}=\frac{EC_{50}^{n}}{C^{n}+EC_{50}^{n}}$ by a non-linear regression (see estimated parameter values in Table S2).

However, since the global blend concentration was the sum of that of its components we could not determine which type of blend interaction occurred between (Z)-3-hexenyl acetate and linalool. To this end, we modeled the background doses-response to pheromone and pheromone salience curves for each of the three background types (Figure 8). Our modeling approach revealed that the parameters of the model curves for the blend were always closer to the estimation for (Z)-3-hexenyl acetate than to linalool with stronger estimated EC50 value and a lower coefficient (Figure 8 and Table S2) confirming (Z)-3-hexenyl acetate salience in the background effects and indicating an hypo-additivity mode of interaction.

### 3.5. Multi Component Blends Reproduced the Activity of Salient Compounds

**Figure 9:**
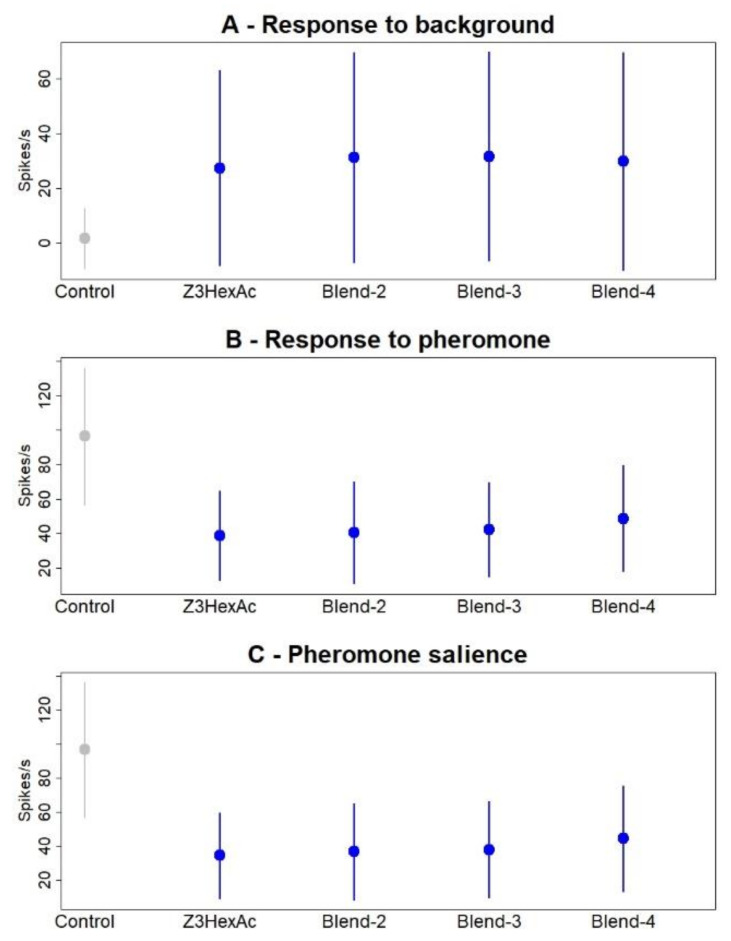
Effects of backgrounds with blends of 3- and 4-components on the Z7-ORN firing activity. Control = mineral oil. Z3HexAc = (Z)-3-hexenyl acetate. Blend-2 = (Z)-3-hexenyl acetate 1 AU plus linalool at ratios of 1:1. Blend 3 = (Z)-3-hexenyl acetate 1 AU plus indole and β-caryophyllene at ratios of 1:1:0.3. Blend 4 (Z)-3-hexenyl acetate 1 AU, linalool, α-pinene and eucalyptol at ratios of 1:1:2:2. (**A**) Response to the background, (**B**) Response to the pheromone, (**C**) Pheromone salience. Means of *n* = 34 measures on 17 Z7-ORNs. Error bars = standard deviations.

Finally, we tested the effects of two more complex backgrounds on pheromone detection by Z7-ORNs. A 3-component background was prepared by mixing the agonist (Z)-3-hexenyl acetate with indole and β-caryophyllene, at a ratio of 1:1:0.3. A 4-component background was prepared by mixing the two agonists, (Z)-3-hexenyl acetate and linalool with α-pinene and eucalyptol, at a ratio of 1:1:2:2. The 2-component background obtained by mixing linalool and (Z)-3-hexenyl acetate was included as a reference. All blends and reference backgrounds were presented at (Z)-3-hexenyl acetate concentration = 1 AU. The 3-component background did not influence neuron firing and response to pheromone any differently from (Z)-3-hexenyl acetate alone (pairwise PERMANOVA, *p* = 0.444, Figure 9B) or the 2-component blend (*p* = 0.908). Similarly, the 4-component background did not influence neuron firing and response to pheromone any differently from either (Z)-3-hexenyl acetate alone (pairwise PERMANOVA, *p* = 0.0088, Figure 9B) or the blend of the two agonists (pairwise PERMANOVA, *p* = 0.0098, Figure 9). No significant difference was observed between the 3- and 4-component blends either (*p* = 0.310).

## 4. Discussion

Our data confirm previous observations that a background of common VPCs interfere with the moth pheromone system in a dose-dependent manner [29,30,62,63]. Interference with the neuronal coding of the pheromone signal starts at the periphery: specialized Ph-ORNs respond to some VPCs and their responses to the pheromone compound Z7-12:Ac are affected by a VPC background. Such interactions between odorants can potentially occur at many levels in the sensillum, which makes difficult to identify their precise mechanisms. In rats, non-competitive interactions resulting in mixture suppressions play a major role in the blend interactions that contribute to the perception of natural odorant mixtures [60]. Competitive binding at the OR level has been firmly established in insects for the HR13 pheromone receptor of *Heliothis virescens* [34]. With electrophysiology we mostly observed a reduced response to the pheromone compound Z7-12:Ac when it was presented over a single-VPC background. By contrast, in Ca-imaging, the response to Z7-12:Ac was slightly increased in a VPC background. This contrasts with studies in bees where Ca-imaging revealed a majority of mixture suppressions in the AL [64], but with a different protocol, COVs being delivered simultaneously to the bee antenna. In the present study while the firing response to VPCs decreased rapidly to a lower level after an initial peak, the calcium-response stayed at the same level for the whole duration of the background application to slowly decrease when it was turned off. Calcium fluorescence has a much slower kinetics compared to firing activity, which makes difficult to interpret it in terms of interaction between odors presented with a delay. Furthermore, to fully explain the decreased response to pheromone it should be noted that in our experiments the Z7-ORNs reached a certain degree of adaptation before the Z7-12:Ac puff because of their response to the VPC background. Thus, in addition to a mixture suppression, cross-adaptation between background and pheromone probably also contributed to reduce the response to pheromone. Whether environmental VPC concentrations can induce similar adaptation remains unknown. 

VPCs differed in their capacity to stimulate Z7-ORNs and such differences cannot be explained by stimulus intensities because we adjusted the source concentrations to vapor pressures. Thus, these differences must be attributed to the binding selectivity of ORs or any other olfactory proteins. Although in the present work the pheromone salience was lower in a background of VPCs activating the Z7-ORNs, other studies revealed that responses to pheromone can also be decreased by VPCs with no intrinsic activity [28]. A clear inhibition of the pheromone response by linalool was observed in vivo in a noctuid moth, *Spodoptera littoralis*, although at the concentrations used linalool did not activate Ph-ORNs (Party, Hanot et al., 2009). This suggests that VPCs can alter the pheromone binding, whether it be by syntopic or allosteric interactions. It makes difficult to predict the impact of one VPC on pheromone detection. Interestingly, we observed large differences between VPCs in the temporal dynamics of the firing responses. A fast response followed by a decrease was observed for (Z)-3-hexenyl acetate, while linalool elicited a delayed increase in firing. Differences in the time-course of odorant concentrations due to air-surface interactions within the system delivering the stimuli have been measured by physical methods in an olfactometer [65,66]. Odorants adsorb to the walls of the stimulator device and desorb later on, resulting in slower stimulus rise and decline. Adsorption depends on the affinity of the odorant molecules for the tubing which explain that stimulus rise and decline times vary notably among compounds. It is not known whether a similar process also exists within olfactory organs and contributes to shape in vivo the stimulus course. In natural conditions, adsorption of volatiles like pheromones onto the vegetation is known to occur [67]. 

In several moth species the integration of pheromonal signal and host plant odors occurs within the male ALs [33,68,69]. Behavioral data confirms that male moths are more attracted in a flight tunnel by blends of pheromone and plant VPCs compared to the pheromone alone suggesting an ecological adaptation [70,71]. However, the other side of the coin is that sexual signals and environmental odorants from host and non-host-plants become mixed. Neuronal treatment of the input generally improves the signal-to-noise ratio in sensory-systems. The olfactory noise in natural environments is not only complex because of the diversity of VPCs but it fluctuates largely independently of the pheromone signal. Such an olfactory noise deteriorates intensity and quality coding of the pheromone signal in laboratory conditions [63]. Furthermore, because of their responses to VPCs, the Ph-ORN outputs may be ambiguous with respect to the nature of the chemical. Does the AL neuronal network facilitate signal identification in downstream olfactory areas by reformatting the ORN input? The high convergence level of many ORNs expressing the same functional type of OR onto a few PNs averages the responses of many ORNs. However, we did not observe any increase in pheromone salience over background from Z7-ORNs to MGC-neurons. The ALs reformat the ORN output, resulting in increased signal-to-noise ratio [72,73]. Convergence should increase pheromone signal to background ratio in PNs, provided not all Ph-ORNs have the same sensitivity to background odorants. However, if most Z7-ORNs respond in a standard way to a background odorant, averaging will not improve signal-to-noise ratio. Then, pooling Ph-ORN outputs might make more apparent among AL neurons the odor stimuli that produce weak ORN responses. To explain this contradiction, it is important to consider that separating signal from noise is not the only challenge of pheromone communication because pheromone concentrations vary over a very large range in natural conditions [74,75]. The dynamic range of moth Ph-ORNs is of seven orders of magnitude while the maximum firing rate of individual neurons is in the range of 150 to 200 spikes per second [16]. Neural gain control mechanisms [23,76] allow the brain to cope with large changes in the level of the ORN input. A number of studies suggests this is a key function of the ALs [77]. Gain control alters the relationships between ORNs and PNs firing in such a way that amplification is high when the ORN firing activity is weak, but low when it is strong. Such a non-linear amplification could also increase the representation in the MGC of weak agonists like VPCs. Thus, in the ALs, the neuronal network might have evolved to increase sensitivity and encode fast changes over a wide range of concentrations, possibly at some cost for qualitative selectivity. 

VPCs are naturally emitted by plants in complex mixtures. While each VPC might individually be present at very low mixing rates within the atmosphere, the total levels of VPCs over a forest commonly reach hundreds of ppb to peak at nearly one ppm [78]. It is thus important to establish whether effects of VPCs are cumulative, or whether VPCs interact with each other, and how much such interactions affect pheromone detection. To understand blend perception, interactions between odorants in mixtures have been intensively analyzed in various organisms, including insects, at the periphery [54] and brain levels [64,79]. In most cases the response to a blend is lower compared to the response to the most active component, an interaction called mixture suppression [80]. However, none of these studies considered the case of a composite background interacting with the detection of an odor signal by narrowly tuned receptor neurons. Since the potential sites of interactions are multiple, interactions within multi-component blends are probably difficult to quantify. Nevertheless, dose-response relationships fitted quite well with classical Hill models, enabling quantitative comparison between blends and single compounds. Our analyses reveal that the complexity of the blend, in terms of its number of components, did not play a prominent role in the interaction with pheromone perception. Comparing three- or four-component blends to binary blends or single compounds indicated that a blend showed the activity of its most active compound. Thus, although the diversity of a background might increase the probability of including a VPC capable of interacting with the pheromone system, chemical diversity of the background does not seem to be a prominent factor per se. 

Insects have evolved their efficient pheromone communication system in the presence of a complex natural background of VPCs [3]. However, among other anthropogenic factors, global warming is significantly affecting plant metabolism so that the emissions of VPCs are modified. Current knowledge on the impact of CO_2_ concentration and temperature elevations on plant physiology indicates a fast global increase in their VPC emissions [81] and significant changes in the pattern of terpenoid release [82]. This increase in atmospheric mixing rate of VPC will change olfactory landscapes, which, as confirmed in our study, might impact pheromone communication. Additionally, host plant location might be affected because it has been shown that the ratio between background and host-plant volatiles alter the response of female moths to their host-plant [83]. In *Manduca sexta* the background has important effects on the moth’s ability to locate its host plant [7]. Interestingly, differences between VPCs in their capacity to interfere with insect olfaction indicate that impact will greatly depend on which VPCs are involved. Stimulation of the emission of indigenous plants, or introduction of novel plants that release salient VPCs, like for instance (Z)-3-hexenyl acetate for *A. ipsilon*, will have a greater impact on insect olfactory communication than botanical changes associated to less potent VPCs. It is thus important to better evaluate the impacts of the insect exposure to various odorscapes. Analyses of interactions at the molecular level, will contribute to better predict the risks. We also need quantitative analyses of odorscapes at fine temporal and spatial scales to better estimate the VPC peak concentrations and exposure durations insects experience. 

## Figures and Tables

**Table 1:** Intensities of calcium responses in the MGC following a 5 s presentation of a background of single VPC or of VPC-background plus 1 s puff of the pheromone component Z7-12:Ac. Means (*n* = 15) and standard deviations (in brackets) of the maximum δF/F. Statistical tests: One-way ANOVA, 1 degree of freedom. Significant differences (*p* < 0.05) appear in bold in the *p* column.

Type of Background	δF/F Mean (SD)		Background vs. Z7-12:Ac in Background		Z7-12:Ac vs. Z7-12:Ac in Background		Z7-12:Ac vs. Background	
	Background	Background + Z7-12:Ac	F	*p*	F	*p*	F	*p*
2-hexenal	0.33	1.27 (0.19)	15.4626	**0.0005**	7.60928	**0.01012**	2.63462	0.11576
	(0.14)							
Linalool	0.48	1.15 (0.19)	7.0363	**0.01301**	5.14534	**0.03121**	0.53650	0.46997
	(0.17)							
(Z)3-hexenyl acetate	0.77	1.22 (0.15)	5.7185	**0.02375**	8.69025	**0.00639**	0.60096	0.44471
	(0.11)							
α-pinene	0.05	0.89 (0.15)	21.8173	**0.00007**	1.61932	0.21365	13.73691	**0.00092**
	(0.1)							
Eucalyptol	0.1	0.93 (0.15)	22.1018	**0.00006**	2.17790	0.15116	12.51131	**0.00143**
	(0.08)							
Indole	0.05	0.64 (0.15)	12.8135	**0.00128**	0.00007	0.99343	18.21651	**0.00020**
	(0.06)							
β-caryophyllene	0.05	0.76 (0.14)	19.2145	**0.00015**	0.47821	0.49493	14.73026	**0.00065**
	(0.09)							
Mineral oil	−0.13	0.69 (0.14)	27.2565	**0.00002**	0.09661	0.75824	27.14252	**0.00002**
	(0.08)							

## Data Availability

Data will be shared upon request to the corresponding author.

## Supplementary Materials

The following are available online at https://www.mdpi.com/article/10.3390/insects12050409/s1, **Table S1**: List of the VPCs used to produce odorant backgrounds with their air–mineral oil partition coefficients (*K_hl_*) and their estimated concentrations inside source headspace and in delivered air for the sources in electrophysiological recordings and Calcium imaging. **Table S2**: Estimation of the concentration at ½ maximum response (*EC*_50_) and coefficient n of the modified Hill equations used for modeling of dose-response relations for response to pheromone and pheromone salience in (Z)-3-hexenyl acetate, linalool or blend backgrounds. **Figure S1**: Diagram of the stimulator devices. (A) Diagram of the device used to create single VPC backgrounds and to deliver pheromone stimulus. In total, eight electrovalves (EV) direct the air flow into an empty vial when non-activated (blue vials, NO = normally open circuit); upon activation, air flow is re-directed toward the source vial (red vials, NC = normally closed circuit). Activation of a valve redirects the air flow from an empty vial toward a source vial so the total air flow in the glass tube is kept constant. Source vials contain only one VPC and each VPC-odorized air lines are separated from each other up to the glass main tube in which the pheromone is delivered. (B) Distal end of the stimulator used in the experiments using backgrounds with two or more VPCs. A low dead-volume manifold has been added to create a mixing chamber for VPCs before their entry in the glass tube. The upstream part (not represented) is identical to A. Line colors indicate when tubing conduct clean air (blue) or odorized air (red). **Figure S2**: Opening two valves simultaneously does not modify the dynamics or the concentration of the signal delivered by each of them. For each pair of valves, one was odorized with one concentration of (Z)-3-hexenyl acetate, the other one was left non-odorized as it contained mineral oil only. Valve pairs are, (A) odorized valve EV1 (mineral oil concentration 0.1% *v*/*v*) and non-odorized valve EV2, (B) EV3 (0.25% *v*/*v*) and EV4, (C) EV5 (0.4% *v*/*v*) and EV6, (D) EV7 (0.5% *v*/*v*) and EV8. See **Figure S1B** for valve references. Time-course of stimulus intensity over 12 successive stimuli, where the odorized valve is open alternatively alone (black lines) or at the same time as the non-odorized valve (grey lines). Grey doted lines mark the average plateau intensity of the strongest and weakest response. The magnitude of the difference relative to average plateau intensity of the strongest signal is indicated. To the right of each graph, the results of an ANCOVA model testing the effect of state of the odorized valve and of rank in the stimulation sequence are given. **Figure S3**: Calcium fluorescence activity maps triggered by Z7-12:Ac or single VPCs in the right antennal lobe of a male *A. ipsilon*. **Figure S4**: Four VPCs, β-caryophyllene, indole, isoprene and α-pinene, neither activated MGC neurons nor modified their responses to the pheromone. Strip charts compare individual neuron firing activities in each VPC background (green dots) with the control background (black dots). The firing frequency of MGC neurons was measured during different time windows to evaluate their response to background (left column), response to pheromone (middle column), and pheromone salience (right column). NS = *p*-value above FDR threshold. *n* = 10. **Figure S5**: Four VPCs, β-caryophyllene, indole, isoprene and α-pinene neither activated Z7-ORNs not modified their responses to the pheromone. Strip charts compare individual neuron firing activities in each VPC background (black dots) with the control background (red dots). Z7-ORN firing frequency was measured during appropriate time windows to evaluate their response to background (left column), response to pheromone (middle column), and pheromone salience (right column). NS = *p*-value above FDR threshold. *n* = 26. **Figure S6**: A puff of eucalyptol has an inhibitory effect on the response to pheromone of Z7-ORNs. (A) Stimulation protocol applied in this experiment. Green and black boxes indicate the delivery of the VPC background and the pheromone on the moth antenna, respectively. TW: limits of the two time-windows used in the data analysis. (B) Comparison of the effects of a VPC puff (green dots) vs. control (red dots) on the pheromone response. *n* = 13. Stars indicate *p*-values of the paired t test below FDR threshold.
