# Supplementary material for: Effects of Multi-Component Backgrounds of Volatile Plant Compounds on Moth Pheromone Perception
Source: Insects. 2021 May 1;12(5):409. doi: 10.3390/insects12050409 (PMC8147264; doi:10.3390/insects12050409)
Supplement: Supplementary file 1 [file insects-12-00409-s001.zip › Supplementary Figure S1-S6.pdf]

Supplementary Figure S1–S6

Repeatability of background concentration during blend experiments

From 2 to 4 valves were activated during blend experiments, each valve controlling one constituent of the blend. The total airflow was unchanged during valve activation. We checked the repeatability of background delivery and made sure that the aerial concentration of each constituent was equal when delivered alone or as part of a blend. To this end, we assigned each valve of the stimulation device (Figure S1-B) to a pair. For each pair, one valve was connected to a vial loaded with a (Z)-3-hexenyl acetate solution (Z3HexAc-valve), the other to a vial loaded with pure mineral oil (MO-valve). We monitored (Z)-3-hexenyl acetate concentration in the glass tube with a photo-ionization detector (miniPID, Aurora Scientific Inc, Aurora, Canada). The input needle of the PID was set at the outlet of the glass tube, centered in its cross-section. For each pair of vials, the Z3HexAc-valve was opened for 5 s, alternatively alone or simultaneously with the MO-valve. This sequence was replicated 6 times for each pair, with 5 min intervals between successive openings of the Z3HexAc valve. An ANCOVA model was used to test whether the state of the MO-valve (open or closed) affected mean plateau signal intensity delivered by the Z3HexAc-valve with the rank in the stimulation sequence as a covariate. PID signal intensity and dynamics as delivered by the Z3HecAc-valve were not altered by the simultaneous opening of the MO-valve (Figure S2, MO-valve state effect: $p > 0.05$ for all four Z3HexAc-valves/concentrations tested). Stimulus intensity did not decrease more than 9% in relative value along the stimulation sequence. We insured to use the same pairs of valves in the binary blend tests.

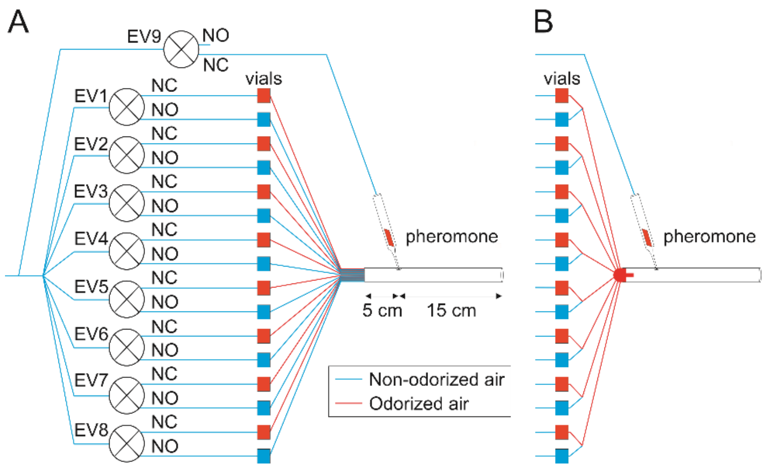

Figure S1: Diagram of the stimulator devices. (A) Diagram of the device used to create single VPC backgrounds and to deliver pheromone stimulus. Eight electrovalves (EV) direct the air flow into an empty vial when non-activated (blue vials, NO = normally open circuit); upon activation, air flow is re-directed toward the source vial (red vials, NC = normally closed circuit). Activation of a valve redirects the air flow from an empty vial toward a source vial so the total air flow in the glass tube is kept constant. Source vials contain only one VPC and each VPC-odorized air lines are separated from each other up to the glass main tube in which the pheromone is delivered. **(B)** Distal end of the stimulator used in the experiments using backgrounds with two or more VPCs. A low dead-volume manifold has been added to create a mixing chamber for VPCs before their entry in the glass tube. The upstream part (not represented) is identical to A. Line colors indicate when tubing conduct clean air (blue) or odorized air (red).

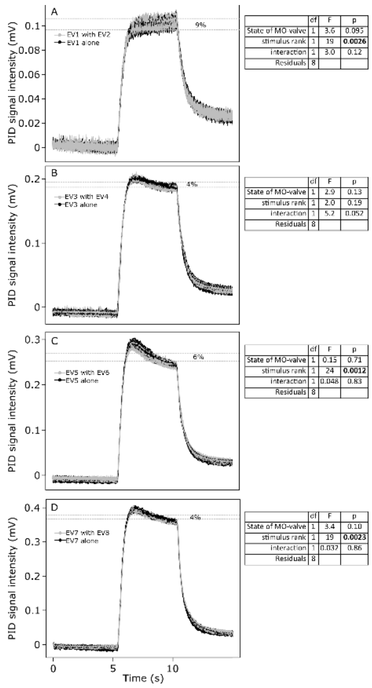

A

	df	F	p
State of MO-valve	1	3.6	0.095
stimulus rank	1	19	**0.0026**
interaction	1	3.0	0.12
Residuals	8		

B

	df	F	p
State of MO-valve	1	2.9	0.13
stimulus rank	1	2.0	0.19
interaction	1	5.2	0.052
Residuals	8		

C

	df	F	p
State of MO-valve	1	0.15	0.71
stimulus rank	1	24	**0.0012**
interaction	1	0.048	0.83
Residuals	8		

D

	df	F	p
State of MO-valve	1	3.4	0.10
stimulus rank	1	19	**0.0023**
interaction	1	0.032	0.86
Residuals	8		

Figure S2: Opening two valves simultaneously does not modify the dynamics or the concentration of the signal delivered by each of them. For each pair of valves, one was odorized with one concentration of (Z)-3-hexenyl acetate, the other one was left non-odorized as it contained mineral oil only. Valve pairs are, A) odorized valve EV1 (mineral oil concentration 0.1 % v/v) and non-odorized valve EV2, B) EV3 (0.25 % v/v) and EV4, C) EV5 (0.4 % v/v) and EV6, D) EV7 (0.5 % v/v) and EV8. See figure S1B for valve references. Time-course of stimulus intensity over 12 successive stimuli, where the odorized valve is open alternatively alone (black lines) or at the same time as the non-odorized valve (grey lines). Grey doted lines mark the average plateau intensity of the strongest and weakest response. The magnitude of the difference relative to average plateau intensity of the strongest signal is indicated. To the right of each graph, the results of an ANCOVA model testing the effect of state of the odorized valve and of rank in the stimulation sequence are given.

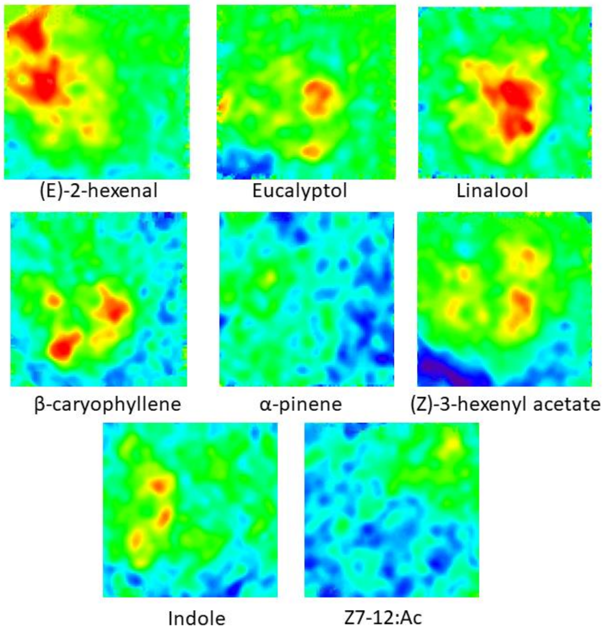

Figure S3: Calcium fluorescence activity maps triggered by Z7-12:Ac or single VPCs in the right antennal lobe of a male *A. segetum*.

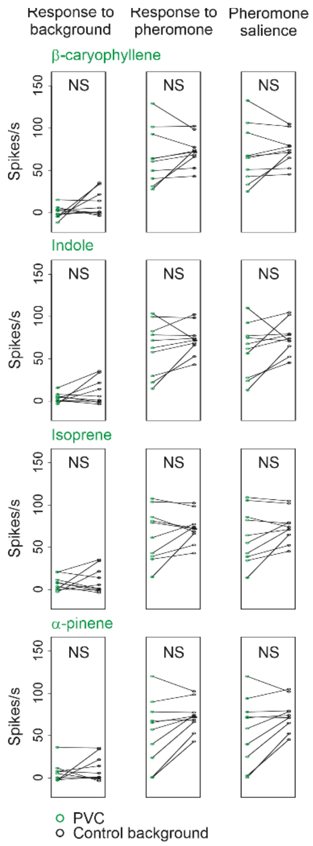

Figure S4: Four VPCs, β-caryophyllene, indole, isoprene and α-pinene, neither activated MGC neurons nor modified their responses to the pheromone. Strip charts compare individual neuron firing activities in each VPC background (green dots) with the control background (black dots). The firing frequency of MGC neurons was measured during different time windows to evaluate their response to background (left column), response to pheromone (middle column), and pheromone salience (right column). NS = p-value above FDR threshold. N = 10.

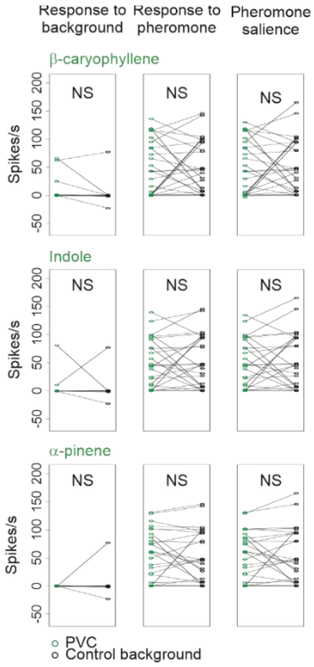

Figure S5: Four VPCs, β-caryophyllene, indole, isoprene and α-pinene neither activated Z7-ORNs not modified their responses to the pheromone. Strip charts compare individual neuron firing

activities in each VPC background (black dots) with the control background (red dots). Z7-ORN firing frequency was measured during appropriate time windows to evaluate their response to background (left column), response to pheromone (middle column), and pheromone salience (right column). NS = p-value above FDR threshold. N = 26.

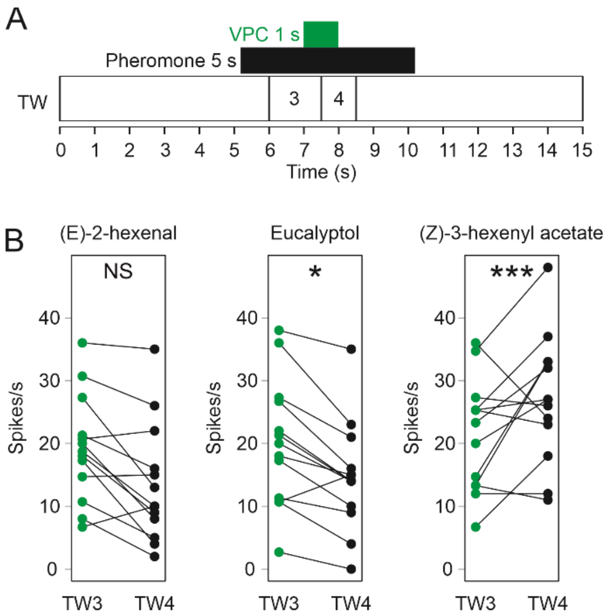

Figure S6: A puff of eucalyptol has an inhibitory effect on the response to pheromone of Z7-ORNs. (A) Stimulation protocol applied in this experiment. Green and black boxes indicate the delivery of the VPC background and the pheromone on the moth antenna, respectively. TW: limits of the two time-windows used in the data analysis. (B) Comparison of the effects of a VPC puff (green dots) vs. control (red dots) on the pheromone response. N =13. Stars indicate p-values of the paired t test below FDR threshold.
