# Supplementary material for: Effects of Multi-Component Backgrounds of Volatile Plant Compounds on Moth Pheromone Perception
Source: Insects. 2021 May 1;12(5):409. doi: 10.3390/insects12050409 (PMC8147264; doi:10.3390/insects12050409)
Supplement: Supplementary file 1 [file insects-12-00409-s001.zip › Supplementary Table-S1-S2.pdf]

Supplementary Table S1–S2

Volatile plant compound	CAS number	Nominal purity (%)	Concentration in mineral oil		Air-mineral oil partition coefficient K_{hl}	Concentration in air	
			% v/v	mol/L		mol/L in closed source headspace	AU delivered
Indole	120-72-9	99	saturated solution	$2.08*10^{-4}$	$1.9*10^{-6}$	$3.9*10^{-10}$	0.9
(E)-2-hexenal	6728-26-3	98	0.1	$8.6*10^{-6}$	$6.3*10^{-4}$	$5.4*10^{-9}$	13.0
Eucalyptol	470-82-6	> 98	1	$6.0*10^{-5}$	$1.5*10^{-5}$	$8.9*10^{-10}$	2.1
α-pinene	80-56-8	98	1	$6.3*10^{-5}$	$3.8*10^{-5}$	$2.4*10^{-9}$	5.7
Linalool	78-70-6	97	1	$5.6*10^{-5}$	$7.5*10^{-6}$	$4.2*10^{-10}$	1.0
Isoprene	78-79-5	> 99.5	0.0001	$1.0*10^{-8}$	$1.1*10^{-2}$	$1.1*10^{-10}$	0.3
β-caryophyllene	87-44-5	> 80	10	$4.4*10^{-4}$	$1.9*10^{-7}$	$8.5*10^{-11}$	0.2
(Z)-3-hexenyl acetate	3681-71-8	> 98	1	$6.3*10^{-5}$	$2.7*10^{-5}$	$1.71*10^{-9}$	4.1

Table S1: List of the VPCs used to produce odorant backgrounds with their air-mineral oil partition coefficients (K_{hl}) and their estimated concentrations inside source headspace and in delivered air for the sources in electrophysiological recordings and Calcium imaging. K_{hl} were measured by injecting the headspace of vials containing appropriate volumes and dilutions of the compounds in mineral oil, equilibrated at 22°C, onto a GC-FID. For compounds with $K_{hl} > 1 *10^{-5}$, we used the Phase Ratio Variation method, keeping the vial volume and the concentration in mineral oil constant while changing the volume of solution (Ettre et al., 1993). For the other compounds, we varied the concentration of the solution introduced in the vial and calibrated the FID detector. The concentration in the headspace (C_h) of the closed equilibrated source was calculated as:

$$C_h = K_{hl} * C_l$$

The delivered concentration also depends on a dilution factor of unknown value, induced by the air flow running through the headspace of the source. We therefore expressed aerial concentrations in a relative, arbitrary unit, where 1 AU is defined as the molar concentration delivered by a source loaded with a solution of linalool in mineral oil at 1% v/v. This was done under the assumption that the dilution factor is concentration- and compound-independent.

Reference

Ettre, L. S., Welter, C., & Kolb, B. (1993). Determination of gas-liquid partition coefficients by automatic equilibrium headspace-gas chromatography utilizing the phase ratio variation method. *Chromatographia*, *35*(1-2), 73-84.

Response to pheromone								
	EC_{50}				n			
		sd	t value	P		sd	t value	P
(Z)-3-hexenyl acetate	0.4654	0.1066	4.364	$4.920*10^{-5}$	0.6585	0.1312	5.018	$4.67*10^{-6}$
Linalool	1.8279	0.7733	2.364	$2.123*10^{-2}$	0.4458	0.1303	3.421	$1.11*10^{-3}$
Blend	0.6427	0.1041	6.174	$5.650*10^{-8}$	0.7339	0.1056	6.950	$2.62*10^{-9}$
**Pheromone salience**								
(Z)-3-hexenyl acetate	0.4228	0.0806	4.913	$6.870*10^{-6}$	0.7790	0.1472	5.293	$1.67*10^{-6}$
Linalool	1.5073	0.5678	2.654	$1.008*10^{-2}$	0.4769	0.1323	3.606	$6.22*10^{-4}$
Blend	0.6001	0.0876	6.852	$3.870*10^{-9}$	0.8519	0.1183	7.201	$9.65*10^{-10}$

Table S2: Estimation of the concentration at ½ maximum response (EC_{50} **) and coefficient n of the modified Hill equations** used for modeling of dose-response relations for response to pheromone and pheromone salience in (Z)-3-hexenyl acetate, linalool or blend backgrounds. Pooled data (n = 16) were fitted to equations $R_{norm}\ or\ S_{norm} = \frac{EC_{50}^{n}}{C^{n}+EC_{50}^{n}}$ (see material and methods) by a non-linear regression.
